# Impact of Quorum Sensing on the Virulence and Survival Traits of *Burkholderia plantarii*

**DOI:** 10.3390/plants13182657

**Published:** 2024-09-23

**Authors:** Minhee Kang, Duyoung Lee, Mohamed Mannaa, Gil Han, Haeun Choi, Seungchul Lee, Gah-Hyun Lim, Sang-Woo Kim, Tae-Jin Kim, Young-Su Seo

**Affiliations:** 1 Department of Integrated Biological Science, Pusan National University, Pusan 46241, Republic of Korea; wertyui@pusan.ac.kr (M.K.); dlendud2164@pusan.ac.kr (D.L.); mannaa@cu.edu.eg (M.M.); croone@pusan.ac.kr (G.H.); hechoi98@pusan.ac.kr (H.C.); dltmdcjf13@pusan.ac.kr (S.L.); ghlim16@pusan.ac.kr (G.-H.L.); kimsw@pusan.ac.kr (S.-W.K.); tjkim77@pusan.ac.kr (T.-J.K.); 2 Institute of System Biology, Pusan National University, Pusan 46241, Republic of Korea; 3 Department of Plant Pathology, Faculty of Agriculture, Cairo University, Giza 12613, Egypt

**Keywords:** *Burkholderia plantarii*, quorum sensing, pathogenicity, survival

## Abstract

Quorum sensing (QS) is a mechanism by which bacteria detect and respond to cell density, regulating collective behaviors. *Burkholderia plantarii*, the causal agent of rice seedling blight, employs the LuxIR-type QS system, common among Gram-negative bacteria, where LuxI-type synthase produces QS signals recognized by LuxR-type regulators to control gene expression. This study aimed to elucidate the QS mechanism in *B. plantarii* KACC18965. Through whole-genome analysis and autoinducer assays, the *plaI* gene, responsible for QS signal production, was identified. Motility assays confirmed that C8-homoserine lactone (C8-HSL) serves as the QS signal. Physiological experiments revealed that the QS-defective mutant exhibited reduced virulence, impaired swarming motility, and delayed biofilm formation compared to the wild type. Additionally, the QS mutant demonstrated weakened antibacterial activity against *Escherichia coli* and decreased phosphate solubilization. These findings indicate that QS in *B. plantarii* significantly influences various pathogenicity and survival traits, including motility, biofilm formation, antibacterial activity, and nutrient acquisition, highlighting the critical role of QS in pathogen virulence and adaptability.

## 1. Introduction

Bacteria utilize a mechanism known as *quorum sensing* (QS) to detect cell density in their environment and regulate gene expression cooperatively. QS influences the transcriptional regulation of various genes involved in processes such as bioluminescence, exoenzymes production, biofilm formation, and pathogenicity [1,2,3]. Understanding QS is crucial for studying the physiology and pathogenicity of bacterial pathogens.

Among the different QS systems, many Proteobacteria use acylated homoserine lactone (AHL) signaling systems, while most Gram-positive bacteria rely on oligopeptide signaling systems [4]. The AHL-mediated QS system is the most common and well-studied in Gram-negative bacteria [5]. In these systems, LuxI-type synthase enzymes produce extracellular signal molecules called autoinducers, which are detected by LuxR-type receptors to assess bacterial density in the surrounding environment [2].

*Burkholderia plantarii*, the causal agent of rice seedling blight, was first identified in 1987 [6]. In South Korea, *B. plantarii* was first reported in 2016 [7]. This pathogen has several virulence factors, with tropolone being a major one, leading to seedling wilt, root growth suppression, and chlorosis [6]. *B. plantarii* is genetically similar to *Burkholderia glumae,* the causative agent of bacterial panicle blight in rice [8]. *B. glumae* has a well-characterized QS system involving the LuxIR-type system. TofI synthesizes QS signal molecules, which are recognized by TofR. Subsequently, QsmR, the *quorum-sensing* master regulator, controls the production of various metabolic products [9,10,11,12]. In 2006, Solis et al. first identified and characterized the QS system in *B. plantarii*, demonstrating that the *plaI* gene, a homolog of the *luxI* family, regulates QS via *N*-acyl homoserine lactones (AHLs). Their study showed that the *plaI* insertion mutant exhibited a significant reduction in virulence in rice seedling blight, establishing the foundational role of QS in the pathogenicity of this bacterium [13].

Genomic studies have provided significant insights into the unique features of *Burkholderia* species, revealing their high genetic versatility and adaptability to various ecological niches [14]. The ability of these bacteria to thrive in diverse environments is attributed to their large multi-replicon genomes, which are rich in insertion sequences and genomic islands. These genomic elements not only enhance their metabolic diversity but also contribute to their ability to adapt to changing environmental conditions. Comparative genomic studies on *B. plantarii* have further emphasized these characteristics, demonstrating the extensive genetic resources this species possesses. This genetic complexity is linked to the presence of numerous virulence factors, which play crucial roles in the pathogenicity of *B. plantarii*. These studies have identified various genes and regulatory mechanisms that enable *B. plantarii* to infect host plants, evade immune responses, and cause disease symptoms. Understanding these genomic traits is essential for developing strategies to manage the diseases caused by this pathogen and for exploring its potential applications in biotechnology and agriculture [15].

Despite the significant impact of *B. plantarii* on rice productivity, comprehensive studies on this pathogen are scarce. To address this gap, an extensive analysis of the *B. plantarii* KACC18965 genome was conducted, comparing it with *B. glumae* BGR1 to identify the gene responsible for QS signal molecule biosynthesis. Various QS-regulated physiological traits, including virulence, motility, biofilm formation, interbacterial interactions, and phosphate solubilization were examined. This study aims to elucidate the QS mechanism in *B. plantarii* and its role in regulating various physiological traits, thus providing a foundation for developing strategies to mitigate its pathogenic effects on rice seedlings.

## 2. Results

### 2.1. Selection of plaI Candidate Genes

To identify the gene responsible for QS signal synthesis in *B. plantarii*, homologous genes of *tofI* in the genome of *B. plantarii* KACC18965 were screened. Three candidate genes, *plaI*, *plaI2*, and *plaI3*, were identified based on their high sequence homology with *tofI* (Table 1). Each of these genes contained motifs associated with AI synthase function. Notably, only *plaI* and *plaI2* were located adjacent to the *tofR* homologous gene (*plaR*), as observed in *B. glumae*.

### 2.2. Gene Involved in Producing a QS Signal Molecule

**Figure 1:**
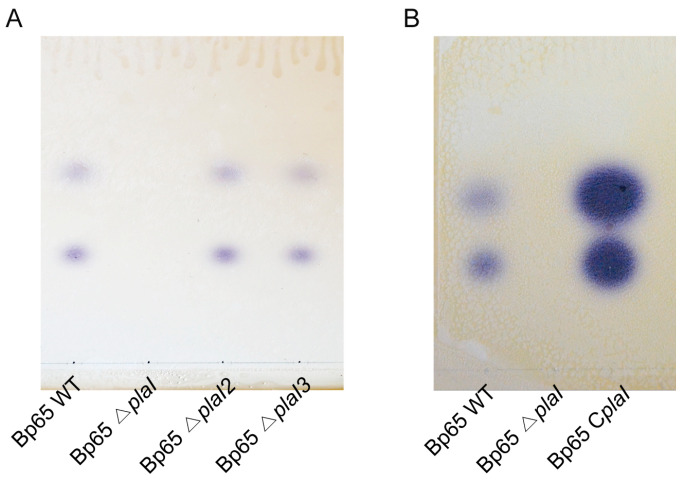
Autoinducers (AIs) production of *B. plantarii* KACC18965 strains. Detection of C8-HSL and C6-HSL was performed using the biosensor strain *Chromobacterium violaceum* on thin-layer chromatography (TLC). (**A**) AI production was undetectable in the *plaI* deletion mutant (Bp65 ∆*plaI*) compared to the wild type (Bp65 WT) and other *plaI* deletion mutants (Bp65 ∆*plaI2* and Bp65 ∆*plaI3*). (**B**) AI production was absent in the *plaI* deletion mutant (Bp65 ∆*plaI*) but was restored in the complemented strain (Bp65 C*plaI*).

LuxI-type synthases are known to produce AHLs, which act as QS signal molecules in Gram-negative bacteria [2]. To confirm which *plaI* gene is involved in AHL production, an autoinducer (AI) thin-layer chromatography (TLC) assay was conducted on *B. plantarii* wild-type and *plaI* deletion mutants [9]. The AI TLC assay revealed the presence of *N*-hexanoyl homoserine lactone (C6-HSL) and *N*-octanoyl homoserine lactone (C8-HSL) in the wild-type, *plaI2* deletion mutant, and *plaI3* deletion mutant, but not in the *plaI* deletion mutant (Figure 1A). To verify that the absence of AHL production was due to the deletion of *plaI*, an AI assay with a *plaI* complemented strain was performed. The complemented strain restored AHL production, confirming that *plaI* is the gene responsible for QS signal synthesis in *B. plantarii* (Figure 1B).

### 2.3. Reduced Virulence in plaI Deletion Mutant

*B. plantarii* is known to cause bacterial seedling blight in rice and can also infect rice during the flowering stage, leading to symptoms in rice panicles. To investigate the role of QS in the virulence of *B. plantarii*, both seedling and flowering tests using the wild type, *plaI* deletion mutant, and *plaI* complemented strain were conducted.

**Figure 2:**
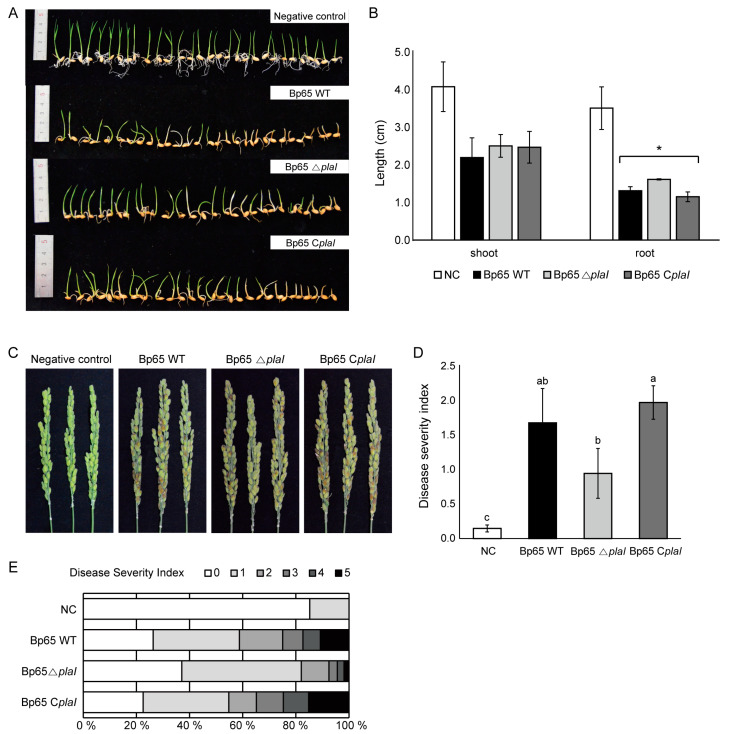
Virulence of *B. plantarii* KACC18965 strains. (**A**) Photographs representing the seedlings infected with *B. plantarii* strains, including the wild type (Bp65 WT), *plaI* deletion mutant (Bp65 ∆*plaI*), and *plaI* complemented strain (Bp65 C*plaI*), compared to a negative control. (**B**) Quantitative analysis of shoot and root lengths of seedlings. Seedlings infected with the *plaI* deletion mutant (Bp65 ∆*plaI*) exhibited longer root lengths compared to those infected with the wild type or *plaI* complemented strain. Significant differences according to LSD test in root lengths are indicated by an asterisk (*, *p* < 0.05). (**C**) Photographs representing the disease symptoms on rice panicles caused by the different *B. plantari*i strains and the negative control. (**D**) Disease severity index of rice panicles infected with the *B. plantarii* strains. The *plaI* deletion mutant (Bp65 ∆*plaI*) showed a lower disease severity index compared to the wild type and *plaI* complemented strain. Different letters on the error bar indicate statistically significant differences between groups according to LSD test at (*p* < 0.05). (**E**) Distribution of disease severity indices among different treatment groups, illustrating the proportion of panicles within each severity category.

In the seedling test, rice panicles were inoculated with *B. plantarii* KACC18965 wild type, *plaI* deletion mutant, and complemented strain. After 7 days, the lengths of the shoots and roots of the seedlings were measured, and the severity of the disease was compared (Figure 2A). The *plaI* deletion mutant exhibited a slight reduction in virulence compared to the wild type and complemented strain. Specifically, the root length was significantly reduced in the wild type and complemented strain compared to the *plaI* deletion mutant, indicating a significant decrease in virulence only in the roots (Figure 2B).

Additionally, in the flowering test, symptoms were observed 1 week after inoculating rice panicles with the wild type, *plaI* deletion mutant, and complemented strain using the dipping method at the flowering stage (Figure 2C). Consistent with the seedling test results, the *plaI* deletion mutant showed reduced virulence compared to the wild type and complemented strain (Figure 2D,E).

### 2.4. QS-Dependent Differences of Collective Movement

**Figure 3:**
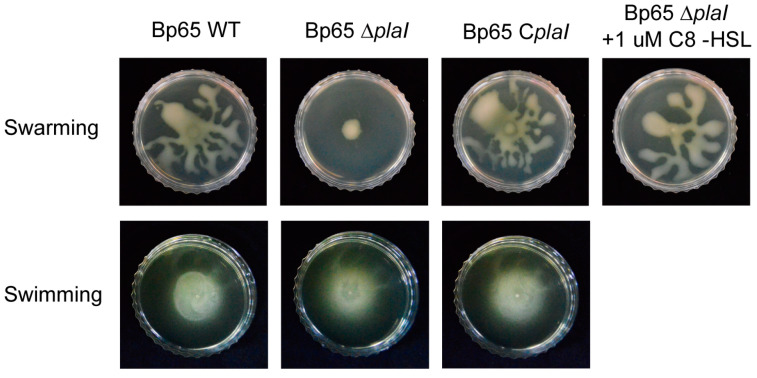
Motility of *B. plantarii* KACC18965 strains. Swarming and swimming motilities of *B. plantarii* strains, including wild type (Bp65 WT), *plaI* deletion mutant (Bp65 ∆*plaI*), *plaI* complemented strain (Bp65 C*plaI*), and *plaI* deletion mutant supplemented with 1 µM C8-HSL. Swarming motility (top row) was significantly reduced in the *plaI* deletion mutant (Bp65 ∆*plaI*) compared to the wild type (Bp65 WT) and the *plaI* complemented strain (Bp65 C*plaI*). The addition of 1 µM C8-HSL to the *plaI* deletion mutant restored swarming motility to the wild-type level. Swimming motility (bottom row) showed no significant difference among the wild type, *plaI* deletion mutant, and *plaI* complemented strain.

Motility is a key mechanism regulated by QS in diverse bacteria [16]. To investigate whether QS influences the collective movement of *B. plantarii*, motility assays were conducted. For individual movement, assessed by swimming assays, all strains displayed similar levels of movement (Figure 3). However, in collective movement, evaluated by swarming assays, the *plaI* deletion mutant exhibited significantly reduced movement compared to the wild type and *plaI* complemented strain (Figure 3). To further identify which AHL acts as the primary QS signal, a swarming assay on the *plaI* deletion mutant supplemented with 1 µM C8-HSL was performed. The addition of C8-HSL restored the swarming ability of the *plaI* deletion mutant to the level observed in the wild type (Figure 3).

### 2.5. QS-Dependent Differences in Biofilm Formation

**Figure 4:**
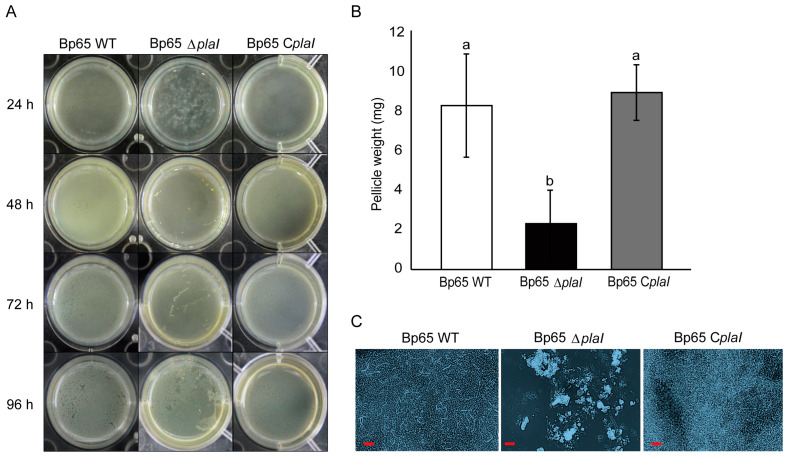
Biofilm formation of *B. plantarii* KACC18965 strains. (**A**) Biofilm formation in static cultures of *B. plantarii* strains over time. Representative images show biofilm formation at the air–liquid interface at 24, 48, 72, and 96 h for the wild type (Bp65 WT), *plaI* deletion mutant (Bp65 ∆*plaI*), and *plaI* complemented strain (Bp65 C*plaI*). The *plaI* deletion mutant exhibited delayed biofilm formation compared to the wild type and complemented strain. (**B**) Quantification of air-dried biofilm mass produced by the tested strains. Bars represent the mean ± standard error of the mean from three independent replicates (*n* = 3). Different letters above the error bars indicate statistically significant differences as determined by the Duncan’s multiple range test (*p* < 0.05). (**C**) Microscopic images of biofilms formed by *B. plantarii* strains 48 h after incubation, stained with safranin. Left: Wild-type (Bp65 WT) biofilm shows a dense, continuous structure. Middle: *plaI* deletion mutant (Bp65 ∆*plaI*) biofilm is fragmented with significantly reduced biofilm mass. Right: *plaI* complemented strain (Bp65 C*plaI*) shows restored biofilm formation similar to the wild type. Scale bar = 10 μm.

To determine the relationship between biofilm formation and QS, *B. plantarii* wild type, *plaI* deletion mutant, and *plaI* complemented strain were cultured under static conditions in PDB. Biofilm formation at the air–liquid interface was observed at 24 h intervals. The wild type and *plaI* complemented strain initiated biofilm formation at 24 h, with the amount of biofilm stabilizing after 48 h. In contrast, the *plaI* deletion mutant began biofilm formation at 72 h, with the amount of biofilm increasing up to 96 h (Figure 4A). To further investigate the impact of QS on biofilm formation, the mass of air-dried biofilms formed at the air–liquid interface after 48 h of static incubation was quantified. The *plaI* deletion mutant exhibited a significantly reduced biofilm mass compared to both the wild type and the complemented strain (Figure 4B). This substantial reduction underscores the critical role of QS in regulating biofilm formation, highlighting the connection between QS and the ability of *B. plantarii* to form robust biofilms under static conditions. Microscopic observations of the biofilms at 48 h post-incubation further supported these findings. The wild type and *plaI* complemented strains displayed dense, continuous biofilm structures, while the *plaI* deletion mutant showed a fragmented and less dense biofilm with scattered cell clusters (Figure 4C). These results highlight the critical role of QS in maintaining biofilm architecture and support the quantitative data, showing that the disruption of the *plaI* gene severely impairs biofilm formation.

### 2.6. Weakened Antibacterial Activity in plaI Deletion Mutant

**Figure 5:**
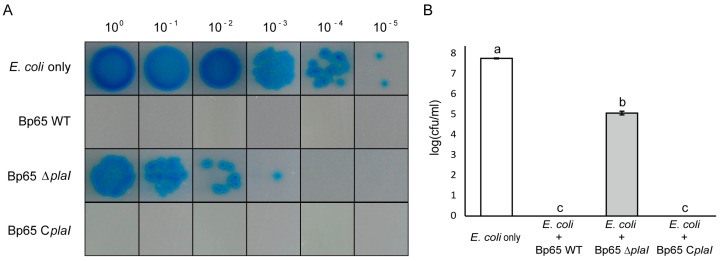
Antibacterial activity of *B. plantarii* KACC18965 strains. (**A**) Co-culture assay showing the survival of *E. coli* when co-cultured with *B. plantarii* strains. Serial dilutions (10^0^ to 10^−5^) of *E. coli* were plated after co-culture with wild type (Bp65 WT), *plaI* deletion mutant (Bp65 ∆*plaI*), and *plaI* complemented strain (Bp65 C*plaI*). *E. coli* co-cultured with the wild type or complemented strain did not survive, while *E. coli* co-cultured with the *plaI* deletion mutant showed significant survival. (**B**) Quantitative analysis of *E. coli* survival in co-culture assays. The log (cfu/mL) values of *E. coli* are shown. *E. coli* only (control) had the highest survival rate, while *E. coli* co-cultured with the wild type or complemented strain had significantly lower survival rates. Different letters on the error bar indicate statistically significant differences between according to LSD test at (*p* < 0.05).

A bacterial competition assay was performed to assess whether QS influences the antibacterial activity of *B. plantarii*. The survival of *E. coli* cells was evaluated after co-culturing with *B. plantarii* wild type, *plaI* deletion mutant, and complemented strain. *E. coli* cells co-cultured with the *B. plantarii* wild-type or *plaI* complemented strain exhibited no survival, as indicated by the absence of colonies. In contrast, *E. coli* cells co-cultured with the *plaI* deletion mutant showed significant survival, demonstrating reduced antibacterial activity in the absence of the *plaI* gene (Figure 5).

### 2.7. Phosphate Solubilization Activity Modulated by QS

**Figure 6:**
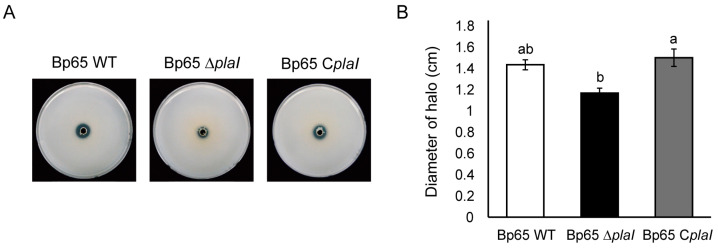
Phosphate solubilizing activity of *B. plantarii* KACC18965 strains. (**A**) Photographs showing the phosphate solubilizing activity of *B. plantarii* strains on Pikovskaya’s agar media. The diameter of the halo around the colonies indicates the extent of phosphate solubilization. The *plaI* deletion mutant (Bp65 ∆*plaI*) formed a smaller halo compared to the wild type (Bp65 WT) and the *plaI* complemented strain (Bp65 C*plaI*). (**B**) Quantitative analysis of the diameter of phosphate solubilization halos. The diameter of the halos was measured, showing that the *plaI* deletion mutant (Bp65 ∆*plaI*) had significantly reduced phosphate solubilizing activity compared to the wild type and complemented strain. Different letters on the error bar indicate statistically significant differences according to LSD test between groups at (*p* < 0.05).

To investigate further activities regulated by QS in *B. plantarii*, the wild type and *plaI* deletion mutant were cultured on various detection media, including siderophore detection plates, skim milk agar for proteinase activity, and oxidative stress tolerance assays. However, none of these tests revealed any significant differences in the deletion mutant. Notably, the diameter of the halo produced by the *plaI* deletion mutant on PKV agar medium was significantly smaller compared to the wild type and complemented strain (Figure 6). This finding indicates that QS plays a critical role in modulating phosphate solubilization activity in *B. plantarii*.

### 2.8. Changes in Expression of T3SS and T6SS Genes Regulated by QS

**Figure 7:**
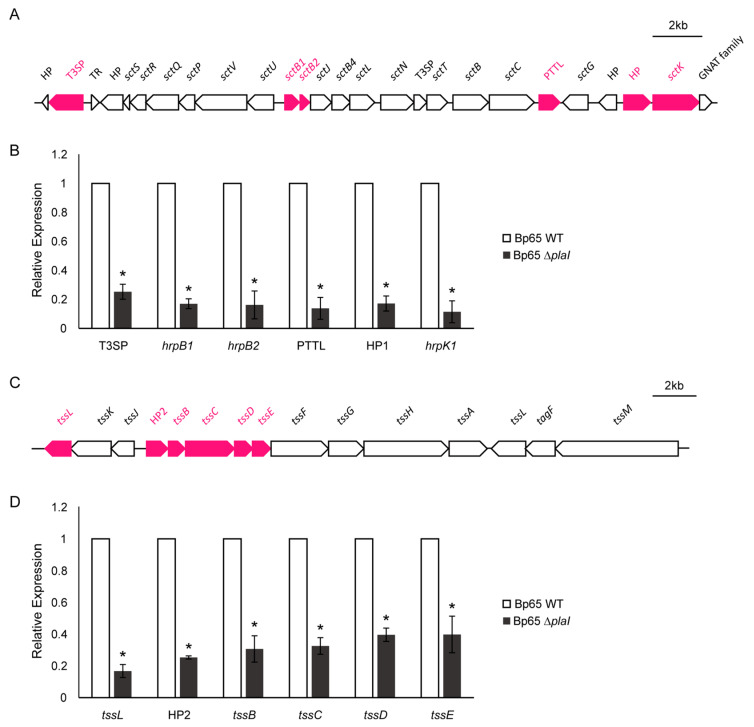
Gene clusters and relative expression levels of T3SS and T6SS genes in *B. plantarii* KACC18965 strains. (**A**) Schematic representation of the *B. plantarii* T3SS gene cluster, highlighting the selected genes analyzed in this study (in red), including *T3SP*, *hrpB1*, *hrpB2*, *PTTL*, *HP1*, and *hrpK1*. (**B**) Quantitative PCR (qPCR) analysis showing relative expression levels of the selected T3SS genes in the wild-type strain (Bp65 WT) and the *plaI* deletion mutant (Bp65 ∆*plaI*). Expression levels of these genes are significantly lower in the *plaI* deletion mutant compared to the wild type. Significant differences in gene expression between the wild type and the mutant are indicated by an asterisk (*, *p* < 0.05). (**C**) Schematic representation of the *B. plantarii* T6SS gene cluster, highlighting the selected genes analyzed (in red), including *tssL*, *HP2*, *tssB*, *tssC*, *tssD*, and *tssE*. (**D**) qPCR analysis demonstrating the relative expression levels of these T6SS genes in the wild type (Bp65 WT) versus the Bp65 ∆*plaI*. Significant differences in gene expression between the wild type and the mutant are indicated by an asterisk (*, *p* < 0.05).

Both Type III Secretion System (T3SS) and Type VI Secretion System (T6SS) are integral to bacterial pathogenicity and antimicrobial activity [17,18,19]. To investigate the role of QS in regulating the T3SS and T6SS, we focused on six key genes from each of these clusters. The qPCR results demonstrated a significant reduction in the expression levels of all selected T3SS and T6SS genes in the *plaI* deletion mutant compared to the wild-type strain (Figure 7). This highlights the crucial role of QS in modulating the expression of T3SS and T6SS genes, which are essential for the pathogenic and interbacterial interactions of *B. plantarii*.

## 3. Discussion

*Quorum sensing* is a crucial communication process in bacteria that allows them to coordinate their behavior by releasing and detecting signal molecules called autoinducers in the environment [3]. QS is integral to various bacterial physiological processes such as virulence, secondary metabolism, DNA competence, and biofilm formation, making it essential for bacterial adaptation and survival in different environments [20,21]. This study focuses on the identification and physiological impacts of QS in *B. plantarii*, a pathogen causing rice seedling blight. The findings of this study support the earlier work by Solis et al. [13], who first demonstrated the role of QS in *B. plantarii* ATCC 43,733 virulence through an insertion mutant of the *plaI* gene. Similar to their findings, a reduction in virulence in the *plaI* deletion mutant was observed, further confirming the importance of QS in regulating pathogenicity. However, the current study extends these insights by exploring additional QS-regulated traits, such as biofilm formation, motility, and antibacterial activity in KACC18965 strain. The deletion mutant in this study displayed a notable reduction in biofilm formation and swarming motility, along with weakened antibacterial activity. These results highlight that QS in *B. plantarii* not only governs virulence but also plays a crucial role in its ecological fitness and survival, particularly in competitive interbacterial interactions.

The current investigation began with identifying the QS signal synthase gene in *B. plantarii*. By analyzing homologous genes to *tofI*, a QS signal synthase in *B. glumae*, three candidate genes (*plaI*, *plaI2*, and *plaI3*) were selected. Deletion mutants for each gene were created, and autoinducer detection assays confirmed that *plaI* functions as the QS signal synthase in *B. plantarii* KACC18965. Furthermore, swarming motility tests showed that the addition of C8-HSL restored the motility of the *plaI* deletion mutant to wild-type levels, indicating that *B. plantarii* uses C8-HSL as a QS signal.

Several studies have shown that QS mutations reduce bacterial virulence by disrupting the regulation of genes critical for pathogenicity and survival in host environments [5]. In our virulence assays, the QS-defective *plaI* mutant of *B. plantarii* exhibited a significant reduction in disease severity compared to the wild type and complemented strain during both the seedling and flowering stages of rice infection. This reduction in virulence is consistent with previous findings that QS is essential for coordinating the expression of virulence factors that enable successful infection and colonization. However, despite the attenuation in virulence, the *plaI* mutant still displayed a considerable level of pathogenicity, suggesting that other virulence mechanisms independent of QS may also contribute to the infection process of *B. plantarii*. This observation is supported by the recent genomic analysis of *B. plantarii*, which identified multiple virulence traits [15]. Additionally, the reduction in root length observed in the seedling test indicates that QS plays a more significant role in root pathogenicity, possibly due to its involvement in regulating factors critical for root invasion and colonization, such as motility, biofilm formation, and the secretion of degradative enzymes. The *plaI* mutant’s reduced ability to form biofilms and its impaired motility further underscore the importance of QS in facilitating the establishment of *B. plantarii* in root tissues.

In motility assays, the degree of swarming in the QS-defective mutant was significantly reduced compared to the wild type, whereas there was no significant difference in the swimming motility between the wild type and the QS-defective mutant. Swarming motility is highly dependent on the QS system because it requires a critical cell density to initiate. Prior research indicates that QS mutations frequently impair swarming in various bacterial species [16]. The observed lack of difference in swimming motility at 28 °C, which is the optimal growth temperature for *B. plantarii*, corroborates findings in *B. glumae* where swimming motility is significantly affected by QS at higher temperatures but not at 28 °C [10]. Additionally, swarming motility has been linked to the invasion and colonization capabilities of pathogens. Studies have shown that reduced swarming motility can lead to decreased virulence, as swarming is essential for effective pathogen dissemination and tissue colonization [10,22]. Therefore, the observed reduction in swarming motility in the QS-defective mutant likely contributes to its decreased virulence, affecting its ability to invade and colonize host tissues effectively.

The biofilm formation assays revealed a notable weight reduction and delay in the initiation and establishment of biofilms in the QS-defective mutant compared to the wild type and complemented strain. This delay is significant as biofilm formation is a crucial factor in the pathogenicity and environmental persistence of bacteria. QS plays a pivotal role in the regulation of biofilm formation across various Gram-negative bacteria, including *Pseudomonas aeruginosa* and members of the *Burkholderia cepacia* complex. These biofilms provide bacteria with enhanced resistance to environmental stressors and antimicrobial agents, thus contributing to their survival and virulence [23,24,25,26]. In *P. aeruginosa*, QS regulates the production of extracellular polysaccharides that form the biofilm matrix, which is essential for the structural integrity and function of the biofilm [23]. This is consistent with our observation of reduced biofilm formation in the QS-defective mutant of *B. plantarii*. Similar findings in *B. cepacia* complex species suggest that QS not only facilitates the initial adhesion and biofilm formation but also influences the development of mature, structured biofilms that are critical for chronic infections [24,25,26]. The delayed biofilm formation observed in the *B. plantarii* QS-defective mutant is likely due to the impaired production of QS-regulated factors that are essential for initiating and maintaining biofilms. The restoration of biofilm formation in the complemented strain further supports the role of QS in this process.

Moreover, the antibacterial activity of *B. plantarii* was assessed by co-culturing it with *E. coli* and it was found that the QS-defective mutant had reduced antibacterial activity. QS is known to regulate antibiotic production in some bacteria [4]. In *B. glumae*, T6SS cluster 1, involved in antibacterial activity, is QS-regulated [27,28]. The findings of the current study suggest a similar QS regulation of antibacterial activity in *B. plantarii.*

Phosphate solubilization assays revealed that the QS-defective mutant had decreased phosphate solubilization ability compared to the wild type. Phosphate-solubilizing bacteria typically dissolve calcium phosphate through gluconic acid-mediated degradation via membrane-bound glucose dehydrogenase (GDH) [29,30]. The reduced phosphate solubilization in the QS-defective mutant indicates QS involvement in this metabolic pathway, although further research is needed to confirm the specific enzymes involved in *B. plantarii.*

Given the established roles of T3SS and T6SS in bacterial pathogenicity and antimicrobial activity in *B. glumae* [17,18,19], their expression in *B. plantarii* was investigated. The qPCR analysis focused on six genes from each secretion system cluster, revealing significant reductions in their expression levels in the QS-defective mutant compared to the wild type. The transcription of T3SS genes, essential for the secretion of virulence factors directly into host cells, has been shown to be regulated by QS in various bacteria, including enteropathogenic *E. coli*, in which the expression of T3SS genes is induced by QS, enhancing the pathogen ability to colonize and cause disease in the host [31]. Similarly, the current study findings indicate that QS positively regulates the expression of T3SS genes in *B. plantarii*. Moreover, QS also modulates the expression of T6SS, which plays a critical role in interbacterial competition and virulence. In *P. aeruginosa*, the QS-dependent transcriptional regulator MvfR positively influences the expression of T6SS *HIS-II* genes [32]. This regulation enhances the bacterium ability to outcompete other microbial species and establish infections. Similarly, the present study shows that the QS system in *B. plantarii* significantly impacts the expression of T6SS genes, thereby influencing its antibacterial activity. In *Aeromonas hydrophila*, QS has been shown to control the secretion of T6SS effectors, which are crucial for the pathogenicity and survival [33]. The results of the current study are consistent with these findings, as diminished antibacterial activity in the QS-defective mutant of *B. plantarii* was observed when co-cultured with *E. coli*. This reduced activity could, at least in part, be related to the role of QS in regulating T6SS and its associated functions in bacterial interactions. Overall, the significant downregulation of T3SS and T6SS genes in the QS-defective mutant of *B. plantarii* highlights the integral role of QS in modulating these critical secretion systems. This modulation is crucial for the pathogen’s ability to infect host plants and compete with other bacterial species, thus contributing to its pathogenicity and ecological fitness.

## 4. Materials and Methods

### 4.1. Bacterial Strains and Culture Conditions

The bacterial strains used in this study are listed in Table 2. The GenBank accession number for *B. plantarii* KACC18965 is GCA_030644525.1. *B. plantarii* strains were grown in Luria-Bertani (LB) broth [1% tryptone (*w*/*v*), 0.5% yeast extract (*w*/*v*), 1% NaCl (*w*/*v*)] and cultured at 28 °C. When necessary, selective antibiotics (100 μg/mL rifampicin; 50 μg/mL kanamycin; 100 μg/mL apramycin) were added to the media.

### 4.2. Construction of B. plantarii plaI Deletion Mutants and Complementation of plaI

Deletion mutants for three *plaI* candidate genes (Δ*plaI*, Δ*plaI*2, and Δ*plaI*3) and complemented strains (C*plaI*) were generated following established protocols. Briefly, standard molecular biology procedures [38] were employed for DNA amplification, recombinant DNA construction, and the generation of mutant strains. For the construction of *plaI* deletion mutants, the upstream (L fragment) and downstream (R fragment) regions of the gene were amplified from *B. plantarii* KACC18965 genomic DNA using Solgent Pfu-X DNA polymerase and primers with appropriate restriction sites. Specific primers were designed for each gene: plaI-LF, plaI-LR, plaI-RF, and plaI-RR for *plaI* deletion; plaI2-LF, plaI2-LR, plaI2-RF, and plaI2-RR for *plaI2* deletion; and plaI3-LF, plaI3-LR, plaI3-RF, and plaI3-RR for *plaI3* deletion (Table S1).

The amplified fragments were ligated into the pK18mobsacB suicide vector, which had been digested with the appropriate restriction enzymes [36]. The recombinant plasmids were first introduced into *E. coli* DH5α λpir cells for replication, then transferred to *E. coli* S17-1 λpir (donor strain), and, subsequently, conjugated into *B. plantarii* KACC18965 (recipient strain) [39]. Deletion mutants were selected using the appropriate antibiotics, and double-crossover recombination was induced by subculturing in LB medium supplemented with 20% sucrose for counter-selection.

For complementation, the full open reading frame of *plaI*, including approximately 273 bp of the upstream promoter region, was cloned into the pBBR1MCS-2 expression vector [37]. The resulting recombinant vectors were introduced into each mutant strain by conjugation. Polymerase chain reaction (PCR) was used to confirm both the deletion mutants and the complemented strains. The strains and plasmids used in this study are listed in Table 2.

### 4.3. Purification and Detection of Autoinducers

The QS signals were purified and detected as described previously [9]. *B. plantarii* KACC18965 wild type, deletion mutants (Δ*plaI*, Δ*plaI2*, Δ*plaI3*), and complemented strain (C*plaI*) were subcultured for 18 h. After mixing the culture medium, excluding the bacteria and an equal amount of ethyl acetate, the supernatant was separated through centrifugation and evaporated to obtain QS signals. The concentrated QS signal samples were developed on Merck silica gel 60 F254 precoated plates with a methanol/water solvent system (70:30, *v*/*v*). After development, the LB agar medium containing the indicator strain *Chromobacterium violaceum* CV026 was placed on a TLC plate and the QS signal was confirmed by reacting for 1 day in a 28 °C incubator.

### 4.4. In Vivo Virulence Assay

To investigate the correlation between QS and virulence in *B. plantarii*, both seedling and flowering tests were conducted using *B. plantarii* KACC18965 wild type, *plaI* deletion mutant, and *plaI* complemented strain as previously described [15].

For the seedling test, 30 Dongjinchal rice panicles (Korea Seed & Variety Service, Republic of Korea) were soaked in 15 mL of bacterial solution at an OD_600_ of 0.8, cultured for 24 h at 28 °C with shaking, and, then, transferred to new containers for germination in an incubator at 28 °C for 3 days. After 3 days of germination, the rice seedlings were cultured for 7 days under conditions of 25 °C for 12 h of daylight and 22 °C for 12 h of night. After 7 days of culturing, the lengths of the shoots and roots were measured.

For the flowering test, Dongjin rice plants were grown at the Department of Southern Area Crop Science of the National Institute of Crop Science (Miryang, Republic of Korea) under greenhouse conditions (32 °C during the day and 25 °C at night). A bacterial suspension with an OD_600_ of 0.8 was inoculated into the flowering-stage Dongjin rice plants using the dipping method for 1 min. After a week, the plants were harvested, and the virulence degree of the panicles was assessed using the following scale: 0—healthy panicle; 1—0 to 20% diseased panicle; 2—20 to 40% diseased panicle; 3—40 to 60% diseased panicle; 4—60 to 80% diseased panicle; and 5—80 to 100% diseased panicle. Disease severity for each plant was calculated using the formula: disease severity = (number of samples per each scale × scale value)/total number of panicles. The virulence assays were repeated in three independent replicates.

### 4.5. Motility Assay

For the motility assays, 1 mL of bacterial culture at an OD_600_ of 0.5 was concentrated to 100 μL. The assays were performed on 0.3% LB agar medium for swimming and 0.5% LB agar medium for swarming. The degree of movement of each sample was observed after 48 h for swimming and 24 h for swarming. The motility assays were repeated in three independent replicates.

### 4.6. Biofilm Formation Assay

For biofilm formation assays, bacteria were cultured for 12 h in potato dextrose broth (PDB) at 28 °C. The cell culture was adjusted to an OD_600_ of 0.05 with PDB, and static culture was carried out in 3 mL volumes in a 12-well plate (SPL Life Sciences, Pochon, Republic of Korea) at 28 °C. Biofilm formation at the air–liquid interface was checked at 24, 48, 72, and 96 h. The biofilm formation assays were repeated with three independent replicates. To further quantify the pellicle biomass, a parallel set of experiments was conducted using larger volumes. Bacterial cultures (OD_600_ = 0.05) were inoculated into 30 mL of PDB in 50 mL glass beakers and incubated under static conditions at 28 °C for 48 h. Following incubation, the pellicle formed at the air–liquid interface was carefully harvested from each replicate. The pellicles were then air-dried for 3 h after all liquid was removed and weighed to quantify the biomass. This experiment was also repeated three times independently to ensure consistent results. Microscopic observations of biofilm formation were conducted after 48 h of static incubation. Biofilms from the wild type, Δ*plaI* deletion mutant, and complemented strains were stained with safranin and observed using an Optinity KB-600F microscope equipped with CoolLED pE300-WHITE illumination (CoolLED). Images were acquired with a KCS3-23S CMOS camera (Korea LabTech, Seoul, Republic of Korea). All images were taken at 100× magnification, and representative images were selected for analysis.

### 4.7. In Vitro Interbacterial Competition Assay

The in vitro interbacterial competition assay was performed as described previously [17]. The *B. plantarii* strains were co-incubated with *E. coli* DH5α harboring the pCRISPomyces-2 plasmid, which confers survival in apramycin and allows alpha complementation for *β*-galactosidase, at a 1:1 ratio on LB agar for 6 h at 28 °C. After collecting the patches cultured together, the number of surviving *E. coli* colonies was measured on LB plates containing apramycin (100 μg/mL) and 5-bromo-4-chloro-indolyl-β-d-galactopyranoside (X-Gal, 40 μg/mL) to confirm the antibacterial activity of *B. plantarii*. The in vitro interbacterial competition assays were repeated with three independent replicates.

### 4.8. Phosphate Solubilization Assay

To assess the phosphate solubilization ability of *B. plantarii* strains, Pikovskaya’s (PKV) agar medium [1% glucose (*w*/*v*), 0.5% Ca_3_(PO_4_)_2_ (*w*/*v*), 0.05% (NH_4_)_2_SO_4_ (*w*/*v*), 0.05% yeast extract (*w*/*v*), 0.02% KCl (*w*/*v*), 0.01% MgSO_4_·7H_2_O (*w*/*v*), 0.0002% MnSO_4_·H_2_O (*w*/*v*), 0.0002% FeSO_4_·H_2_O (*w*/*v*), 1.5% agar (*w*/*v*)] was used. Twenty microliters of culture fluid from each strain were dropped in the center of PKV agar and cultured in an incubator for 5 days at 28 °C. The diameter of the halo zone was then measured. The phosphate solubilization assays were repeated with three independent replicates.

### 4.9. RNA Extraction, and qPCR

The wild-type *B. plantarii* KACC18965 and *plaI* deletion mutant strains were cultured in LB medium at 28 °C for 10 h. Total RNA was extracted using the RNeasy Mini Kit (Qiagen, Valencia, CA, USA). Complementary DNA (cDNA) synthesis and quantitative PCR (qPCR) were performed using an Enzynomics kit according to the manufacturer’s protocol (Enzynomics, Daejeon, Republic of Korea). Primers listed in Table S2 were utilized for qPCR to quantify gene expression levels, and the 16S rRNA gene was used as a housekeeping gene for qPCR with 785F and 907R universal primers [40]. This study focused on the T3SS and T6SS, given their roles in pathogenicity and antibacterial activity. Following the identification and comparison of gene clusters with previous research, we selected six genes from each cluster for detailed analysis. The primary objective was to determine whether these two critical secretion systems are regulated by QS. To this end, the gene expression levels of the selected genes using qPCR was measured, aiming to assess the influence of QS on the regulation of T3SS and T6SS.

### 4.10. Statistical Analysis

Statistical analyses were carried out using the Statistical Analysis Systems (SAS). For the data analysis, variance was assessed using the General Linear Model (GLM) procedures. Mean comparisons were conducted using Duncan’s multiple range test or the least significant difference (LSD) test at a significance level of *p* < 0.05.

## 5. Conclusions

The current study demonstrates that the QS system in *B. plantarii*, which uses C8-HSL as a signal molecule, regulates various physiological traits related to pathogenicity and survival, including virulence, motility, biofilm formation, antibacterial activity, phosphate solubilization, and secretion system expression. This research enhances the understanding of *B. plantarii* pathogenicity and group behavior, providing a foundation for developing targeted strategies to manage rice seedling blight. This study provides a comprehensive understanding of the QS mechanism in *B. plantarii*, offering valuable insights into its group behavior and pathogenicity. These findings lay the groundwork for developing targeted strategies to manage rice seedling blight and exploring potential biotechnological applications. 

## Figures and Tables

**Table 1:** Characteristics of *plaI* genes in *B. plantarii* KACC18965.

Locus Tag	Gene Name	NCBI Function	Identity	Coverage	E-Value
GIY62_33880	*plaI*	GNAT family N-acetyltransferase	99.84	100	0
GIY62_30580	*plaI2*	Cation transporter	99.57	100	0
GIY62_14315	*plaI3*	Hypothetical protein	99.83	100	0

**Table 2:** Bacterial strains and plasmids used in this study.

Bacterial Strain	Description	Source
*Escherichia coli*		Lab collection
DH5α λpir	F^−^ 80d*lacZ*△M15 (*lacZYA-argF*) U169 *recA1 endA1hsdR17* (rk-, mk+) *phoAsupE44-thi-1 gyrA96 relA1*	[34]
S17-1 λpir	*hsdR recA* pro RP4-2 (Tc::Mu; Km::Tn7) (λpir)	
*Chromobacterium violaceum*		[35]
CV026	Autoinducer indicator strain	
*Burkholderia plantarii*		KACC
KACC18965	Wild type	This study
KACC18965 △*plaI*	*B. plantarii* KACC18965 derivative, deletion of 503 bp within GIY62_33880	This study
KACC18965 △*plaI2*	*B. plantarii* KACC18965 derivative, deletion of 490 bp within GIY62_30580	This study
KACC18965 △*plaI3*	*B. plantarii* KACC18965 derivative, deletion of 484 bp within GIY62_14315	This study
KACC18965 C*plaI*	*B. plantarii* GIY62_33880 deletion mutant containing pCplaI	This study
Plasmids		
pK18*mobsacB*	Allelic exchange suicide vector, *sacB* Km^R^	[36]
pDplaI1	pK18*mobsacB*::LR fragment of GIY62_33880 restricted by BamHI and HindIII	This study
pDplaI2	pK18*mobsacB*::LR fragment of GIY62_30580 restricted by BamHI and HindIII	This study
pDplaI3	pK18*mobsacB*::LR fragment of GIY62_14315 restricted by BamHI and HindIII	This study
pBBR1MCS2	Broad-host-range plasmid, Km^R^, for construction of complemented strain	[37]
pCplaI	pBBR1MCS2::CDS of GIY62_33880 and 273 bp upstream sequence of GIY62_33880	This study

Abbreviations: Rif^R^—rifampicin resistance; Km^R^—kanamycin resistance.

## Data Availability

The data that support the findings of this study are available within the paper and its Supplementary Materials online.

## Supplementary Materials

The following supporting information can be downloaded at: https://www.mdpi.com/article/10.3390/plants13182657/s1, Table S1: Primers for mutant construction used in this study; Table S2: Primers for qPCR used in this study.
