# Supplementary material for: Impact of Quorum Sensing on the Virulence and Survival Traits of Burkholderia plantarii
Source: Plants (Basel). 2024 Sep 23;13(18):2657. doi: 10.3390/plants13182657 (PMC11434762; doi:10.3390/plants13182657)
Supplement: Supplementary file 1 [file plants-13-02657-s001.zip › plants-3185960-supplementary.pdf]

Supplementary Materials

Table S1. Primers for mutant construction used in this study

Name	Sequence
plaI-LF	TTTGGATCCTGCCCTGTCAAGGTTGCTA
plaI-LR	GACGCGCAGAGCTGCCTCGTGAACGGAAG
plaI-RF	CCGTTCACGAGGCAGCTCTGCGCGTCGCAAC
plaI-RR	TTTAAGCTTGCACCAACGACATCGAGTT
plaI2-LF	TTTGGATCCCCCGGTTCTACGTATCGTTG
plaI2-LR	ACTGCAGCAGCGGCTTATGGGGTTCCTCGAAAGG
plaI2-RF	TCGAGGAACCCCATAAGCCGCTGCTGCAGTGAAG
plaI2-RR	TTTAAGCTTGATGGCCGAACTGCTGGCAC
plaI3-LF	TTTGGATCCAAAAATGGCCCGGAAAAG
plaI3-LR	ACGTACAGCGAGAACCGTTCGAACGATGCACGACTAC
plaI3-RF	TCGTGCATCGTTCGAACGGTTCTCGCTGTACGTATTC
plaI3-RR	TTTAAGCTTTCAGCTTCCGCAGCTACC

Table S2. Primers for qPCR used in this study

Gene annotation		Sequence
T3S protein	F	TACGCCAAGGATTTCAACGA
(GIY62_29595)	R	GAGTTCGTTGGTCGACGG
hrpB1 (GIY62_29645)	F	ATACCGTCTCGCTGGTGAA
	R	CAGTTCCTCGTTCAGTTCCT
hrpB2 (GIY62_29650)	F	AGATGAGCCTCAACGAGATG
	R	TTTTTCATCAGCGTTTCGAT
Phage tail tip lysozyme	F	AGGTGCAGTTCATGATCCAG
(GIY62_29695)	R	GTTGCGGTTCTGCATTTC
Hypothetical protein	F	GGCCTACTTCCAGCAACAG
(GIY62_29710)	R	GTCTCGATGGCCTTGTAGG
hrpK1 (GIY62_29715)	F	CAACATCGACAACTTCCTGA
	R	GAGTAGTTCTGCGGGTTCTG
tssL (GIY62_12080)	F	GGCGAGAAGTTCTTCCAGTA
	R	GTCAGGTAGGCGAGCTTCTC
Hypothetical protein	F	CCAGATCCAGTTCCAGCA
(GIY62_12095)	R	CAGCACGCTCTTCGACTG
tssB (GIY62_12100)	F	CGATCGAGGTGAAGGAACT
	R	CGATCGATGTTGACGAACTT
tssC (GIY62_12105)	F	GCAGAAGCCCAAGAAATACA
	R	CATCATCGCCTTCAGGTAGT
tssD (GIY62_12110)	F	CTGGATCGAACTCAAGTCGT
	R	CTCCTTCGTGAACACCATGT
tssE (GIY62_12115)	F	GTTCATCTGCAAGTCGATCC
	R	AGTTCAACGAGTTGGTCGAT
